# Psychometric Network Analysis of the Hungarian WAIS

**DOI:** 10.3390/jintelligence7030021

**Published:** 2019-09-09

**Authors:** Christopher J. Schmank, Sara Anne Goring, Kristof Kovacs, Andrew R. A. Conway

**Affiliations:** 1 Department of Psychology, Claremont Graduate University, Claremont 91711, CA, USA; sara.goring@cgu.edu (S.A.G.); andrew.conway@cgu.edu (A.R.A.C.); 2 Institute of Psychology, ELTE Eotvos Lorand University, 1053 Budapest, Hungary; kristof340@googlemail.com

**Keywords:** intelligence, Process Overlap Theory, psychometric network analysis, latent variable modeling, statistical modeling

## Abstract

The positive manifold—the finding that cognitive ability measures demonstrate positive correlations with one another—has led to models of intelligence that include a general cognitive ability or general intelligence (*g*). This view has been reinforced using factor analysis and reflective, higher-order latent variable models. However, a new theory of intelligence, Process Overlap Theory (POT), posits that *g* is not a psychological attribute but an index of cognitive abilities that results from an interconnected network of cognitive processes. These competing theories of intelligence are compared using two different statistical modeling techniques: (a) latent variable modeling and (b) psychometric network analysis. Network models display partial correlations between pairs of observed variables that demonstrate direct relationships among observations. Secondary data analysis was conducted using the Hungarian Wechsler Adult Intelligence Scale Fourth Edition (H-WAIS-IV). The underlying structure of the H-WAIS-IV was first assessed using confirmatory factor analysis assuming a reflective, higher-order model and then reanalyzed using psychometric network analysis. The compatibility (or lack thereof) of these theoretical accounts of intelligence with the data are discussed.

## 1. Introduction

One of the most replicated findings in psychological science is the positive manifold. The positive manifold refers to the finding that all cognitive ability measures tend to be positively correlated. Individuals who score above average on one test of cognitive ability (e.g., verbal reasoning) tend to score above average on other tests of cognitive ability (e.g., numerical reasoning). Beginning with Spearman [1], the positive manifold has been explained by submitting test scores to factor analysis and extracting a single common factor, *g*. The most common interpretation of *g* is that it reflects a general cognitive ability that is variable among people and significant across different tasks. General cognitive ability is a psychological attribute that explains subject differences in cognitive performance (e.g., speed, accuracy) and has been widely accepted in psychological science [2,3]. However, it has also been controversial.

The theory of general intelligence or *g*-theory interprets the general factor of intelligence as general intelligence or general cognitive ability. This means that it interprets *g*, a psychometric construct, as a within-subject psychological phenomenon. Under this framework, *g* is responsible for individual performance, ability, and covariance demonstrated between tasks. Thus, the higher one scores on *g* the better their performance, and these benefits transfer across various tasks or cognitive tests. Therefore, *g* has a causal effect on ability measured by test performance. However, *g* is a mathematically necessary consequence of the positive manifold [4] but not a necessary explanation. Additionally, and importantly, no psychological basis of *g* has been identified for more than a century [5,6]. 

A new approach, Process Overlap Theory (POT; [5]), challenges this view of intelligence, specifically the interpretation of *g* as general cognitive ability or general intelligence. According to POT, the positive manifold does not reflect general cognitive ability. According to POT, there is no such thing as general cognitive ability. POT proposes that the pattern of overlap of generalist (mostly executive) processes with different (spatial, verbal, etc.) specific processes causes the positive manifold. Under this framework, there is no unitary cause of the covariance between tests or test performance, there are multiple causes, some more general and some more specific. In other words, *g* does not explain the positive manifold, the overlap between processes does. Moreover, the same processes tend to be tapped by several factors (hence the overlap) which explains why latent variables in traditional factor models are not process pure. Thus, POT proposes correlated specific abilities are explained without general mental ability or *g*. Accordingly, the general factor is an emergent property: the consequence rather than the cause of correlated performance. 

POT is incompatible with reflective, higher-order latent variable models of intelligence like *g*-theory. Instead, POT proposes that *g* is a formative construct, i.e., the common consequence rather than the common cause of the correlations between tests. There are two ways to model this formative relationship: (a) using latent variable modeling or (b) psychometric network modeling. In previous work, the structure of POT has been demonstrated via latent variable modeling [5]; in the current project, we will pursue the underlying structure of intelligence data using psychometric network modeling. Therefore, with the aim of understanding intelligence at the process level, an exploratory psychometric network model will be conducted as an alternative technique to estimate the underlying structure of intelligence data assuming the POT framework. 

In the current study, we conduct traditional latent variable models and psychometric network models of intelligence using data from the Hungarian Wechsler Adult Intelligence Scale Fourth Edition (H-WAIS-IV; [7]). First, a traditional example of latent variable modeling is presented and key advantages and disadvantages of this model are considered. Next, psychometric network analysis is presented as a novel alternative to latent variable modeling. This is followed by a discussion of a recent publication that directly compares a psychometric network model to a nested latent variable model [8]. Finally, using similar logic to Kan, van der Maas, and Levin [8], the model fit indices of the psychometric network and latent variable models of H-WAIS-IV data were acquired and presented. The compatibility of these results will be discussed assuming either the reflective, higher-order or formative, overlapping model. 

### 1.1. Higher-Order Latent Variable Model of Intelligence

Latent variable modeling allows researchers to explain the covariation among many observed variables and explore the underlying structure of data using multiple unobserved variables [9,10,11]. These models ought to be approached with a specific goal in mind. On the one hand, a researcher with the goal of data exploration or theory generation would benefit from the data-driven techniques of exploratory factor analysis. On the other hand, a researcher with the goal of data or theory validation would benefit from the data-confirming techniques of confirmatory factor analysis. In fact, it was Spearman’s original use of factor analysis in 1904 that began and led to the overall acceptance of modeling intelligence using higher-order and reflective latent variable models [1].

#### 1.1.1. Advantages of Latent Variable Models

One advantage of latent variable modeling is that it allows for the determination or confirmation of the underlying structure of covariation among observed variables. A second advantage of latent variable modeling is that the technique reduces large datasets into fewer and more easily interpretable numbers of unobserved latent factors [12]. For instance, McGrew [13] conducted latent variable modeling on the Wechsler Adult Intelligence Scale Third Edition that consisted of 15 subtests. Following latent variable modelling, five latent factors were extracted that sufficiently explained the covariation among the original 15 observations, achieving data reduction by 67%; it simultaneously established the underlying latent structure of intelligence data. A third advantage of latent variable modeling is that latent factors lack measurement error as a consequence of not being directly measured during data collection. Thus, if multiple latent factors can be estimated, then relationships among them can be measured without error. Finally, the technique allows for the improvement of questionnaires or cognitive test batteries used to measure these unobserved, latent factors. By using previously established latent variable models or theoretically motivated factor structures, items can be assessed for how well they map onto these latent factors. In summary, latent variable models explore or confirm the underlying factor structure of data in an attempt to explain patterns of covariation among observed variables by latent factors. Although latent variable modeling has well-established advantages, several disadvantages specifically related to intelligence and intelligence modeling exist and are discussed next. 

#### 1.1.2. Disadvantages of Latent Variable Models

**Figure 1:**
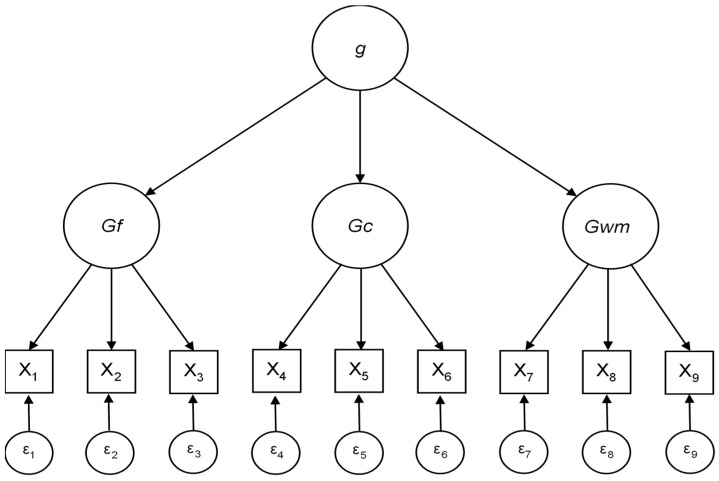
Example latent variable model: Higher-order model of intelligence based on Cattell–Horn–Carroll hierarchical model of general intelligence. Adapted from “Human cognitive abilities: A survey of factor-analytic studies” by Carroll [18]. Circles represent latent variables: general cognitive ability (*g*), fluid intelligence (*G*_f_), crystallized intelligence (*G*_c_), and working memory (*G*_wm_). Smaller circles presented at the bottom of the display represent measurement error (ε_i_) or random noise not explainable by latent variables.

There are two major disadvantages of latent variable modeling. First, once the latent factor structure has been established following exploratory factor analysis, a lack of consensus on how latent factors should be defined and interpreted can occur [14,15,16]. Further, operational definitions of latent factors tend to be subjective because the researcher poses what latent factors represent (for more information on this limitation, see Bock, Goode, and Webb [17]). Additionally, a posteriori or data-driven latent variables might be sample dependent and not truly representative of a specific psychological attribute [14]. This disadvantage does not apply to confirmatory factor analysis nor does it apply to other data-confirming techniques. Data-confirming techniques are theory-based and thus should occur for theoretically motivated factors or variables. As an example of a theoretically motivated factor structure, consider the Cattell–Horn–Carroll (CHC) model of intelligence, as seen in Figure 1) [18,19,20]). Higher-order, or hierarchical, models like this theoretically imply that scores on observed intelligence variables are directly influenced by ability-specific cognitive processes that are each, in turn, influenced by general cognitive ability. These patterns of influence lead us to a more specific disadvantage of reflective and higher-order latent variable models.

The principle of local independence is the second disadvantage of latent variable modeling, specific to assuming a reflective, higher-order model. Assuming these reflective latent variable models implies a common cause of related observations; accordingly, any covariance among observations is fully explained by the latent variable. In a latent variable model, this implies that the observations are locally independent from one another [15]. For example, observations of cognitive ability such as vocabulary, reading comprehension, and listening comprehension would be explained by a single latent variable called verbal ability. The principle of local independence implies that verbal ability accounts for all the shared variance among these cognitive tasks and that no other relationships or shared variance exists among these observations; these variables are ostensibly independent (for more information regarding comorbidity and latent variable models, see Cramer, Waldorp, van der Maas, and Borsboom [21] as well as McNally [22]). As a result, once manifest variables are explained by a latent variable, they pose neither direct nor indirect effects on one another.

#### 1.1.3. Interim Summary

Historically, latent variable modeling has been widely applied in psychological research. However, two main problems persist. First, factors extracted by exploratory factor analysis tend to be subjective in nature. Second, researchers cannot be sure that latent variables directly map onto real psychological attributes. Recently, researchers have begun to pursue topics traditionally explored using latent variable modeling from a different perspective known as psychometric network analysis, e.g., depression [23,24], and post-traumatic stress disorder [25,26].

### 1.2. Psychometric Network Analysis

Researchers using psychometric network analysis assess observed variables and the estimated partial correlations among these observations, without assuming latent common causes. In latent variable modeling, unobserved latent factors are estimated from the variance-covariance patterns among observed variables. On the other hand, psychometric network analysis conceptualizes complex psychological attributes or behaviors as interconnected networks. In terms of network science and network modeling, observations are referred to as nodes and the connections between pairs of nodes are referred to as edges. More recently, Kan et al. [8] provided a description of the differences between traditional latent variable modeling and psychometric network analysis. Kan proposed that psychometric network analysis lends itself to theories of intelligence like POT [5] and their model of intelligence, known as mutualism [27], because these theoretical models of intelligence imply that cognitive processes and abilities interact dynamically with one another. Additionally, Kan [8] provided evidence that, when compared with latent variable models, psychometric network models fit intelligence data better; Kan substantiated this claim by proposing that higher-order latent variable models of intelligence are nested within psychometric network models. 

#### 1.2.1. Advantages of Psychometric Network Analysis

Like latent variable modeling, one advantage of using psychometric network analysis is that it is an exploratory estimation technique that can be used to assess the underlying interconnectedness of observed data. Unlike latent variable modeling, psychometric network analysis completes this task without assuming the presence of unobserved latent factors or constraints from the principle of local independence. Because psychometric network analysis is a statistical technique geared towards data-exploration, a second advantage is that it can guide and inform statistical techniques geared towards data-validation. In essence, psychometric network analysis is similar to exploratory factor analysis. Both statistical techniques focus on the exploration of the covariance between observations, and both techniques generate some form of structure based on these covariances. Third, from a theoretical standpoint, because psychometric network analysis estimates and plots the overlap among observed variables, this statistical technique is primarily adept at modeling intelligence data assuming POT as a theoretical model.

#### 1.2.2. Disadvantages of Psychometric Network Analysis

There are three disadvantages to consider when conducting a psychometric network analysis. First, network models can be misrepresented if observations were collected with a high amount of measurement error. As with latent variable modeling it is important to consider the quality of the data being modeled. Second, psychometric network modeling will only be successful when underlying correlation or covariances are relatively large. For modeling intelligence data, this may not be a problem because the positive manifold tends to be very robust; however, other avenues for research might find network analysis unable to estimate partial correlations that are statistically significantly different from zero. Finally, psychometric network analysis is relatively new to the field of cognitive psychology and intelligence modeling. No standardized procedure has been established across both subfields. Presently, only two publications exist that use psychometric network analysis on intelligence data [8,28].

### 1.3. The Current Project

Using modified R code originally provided by Kan et al. [8] and van der Maas, Kan, Marsman, and Stevenson [28], as well as guidelines established by Epskamp and Fried [29], we assessed the overall fit of H-WAIS-IV data using both latent variable modeling (e.g., higher-order *g* model) and psychometric network analysis. First, confirmatory factor analyses were conducted using the CHC higher-order model of intelligence as a measurement model. Next, psychometric network analyses were conducted. Finally, the model fit indices extracted from both techniques were observed to infer how the H-WAIS-IV data fit assuming either a higher-order latent variable model or a psychometric network model.

## 2. Methods 

### 2.1. Subjects

Sample consisted of 1112 people between 12 and 90 years of age (*M* = 45.15; *SD* = 22.85; *N* = 1110; 646 women). Subjects’ reported level of education indicated that 36.60% had completed primary school, 23.47% had completed some vocational training, 14.30% had completed a college or university degree, 12.95% had completed grammar school (one version of high school in Hungary), 10.43% had completed vocational school (another version of high school in Hungary), and 2.25% had failed to complete primary school. The sample is representative of the population of Hungary in terms of age, geographical location, type of settlement, education, and gender according to the latest census conducted before the standardization of the WAIS.

### 2.2. Measures

The H-WAIS-IV consisted of 15 subtests which may be described as information, vocabulary, comparisons, similarities, picture completion, block design, figure weights, matrix reasoning, visual puzzles, arithmetic, digit span, letter-number sequencing, cancellation, coding, and symbol search. Information about these measures can be found in the Technical and Interpretative Manual [7]. 

### 2.3. Statistical Procedure

#### 2.3.1. Model Fit Evaluation for Latent Variable and Psychometric Network Models

A general approach to model fit evaluation was followed as provided by Kline [10] to assess both psychometric network models and latent variable models. Additionally, due to the continuous nature of the H-WAIS-IV data, model fit evaluation was conducted using suggestions by Hu and Bentler [30] and cutoff criteria published by Schreiber, Stage, King, Nora, and Barlow [31]. Thus, model fit will be deemed appropriate when (a) the ratio of model chi-square (χ^2^) to degrees of freedom is less than or equal to 3.00, (b) comparative fit indices (e.g., Comparative Fit Index (CFI) and Tucker–Lewis Index (TLI)) greater than or equal to 0.95, and (c) Root Mean Square Error of Approximation (RMSEA) values less than or equal to 0.06. Additionally, Akaike Information Criteria (AIC) and Bayesian Information Criteria (BIC) values can be used to compare models: smaller values indicate better fit. 

#### 2.3.2. Confirmatory Factor Analysis

Factor analyses were conducted to assess the model fit of the H-WAIS-IV assuming a higher-order factor model. This model implied six latent variables hierarchically arranged, with one superordinate second-order latent variable representing *g*, and five subordinate first-order latent variables representing crystallized intelligence (*G*_c_), fluid reasoning (*G*_f_), visuospatial ability (*G*_v_), working memory (*G*_wm_), and processing speed (*G*_s_). Measures associated with crystallized intelligence demonstrated excellent test-retest reliability (*r_G_*_c_ = 0.81–0.93). Measures of fluid reasoning, visuospatial ability, working memory, and processing speed all demonstrated medium-to-high test-retest reliabilities (*r_G_*_f_ = 0.70–0.85; *r_G_*_v_ = 0.57–0.81 *r_G_*_wm_ = 0.70–0.89; *r_G_*_s_ = 0.67–0.86; c.f., Sattler & Ryan [32] p. 38). Latent variable models were conducted using the lavaan [33] and openMx [34] packages in R [35] and were visualized using Ωnyx [36]. For access to the R-script of this project see the following OSF project page: https://osf.io/j3cvz/.

#### 2.3.3. Psychometric Network Analysis

Following the latent variable analyses, psychometric network analyses were conducted on correlation matrices extracted from the H-WAIS-IV data. Psychometric network analysis was conducted using the qgraph [37] and openMx [34] packages in R and were visualized using qgraph. Both packages were used to replicate the statistical procedures where psychometric network analysis was conducted on intelligence data presented by van der Maas et al. [28] and more recently by Kan et al [8]. 

Guidelines provided by Epskamp and Fried [29] were followed when conducting psychometric network analysis. Network models were generated using the graphical least absolute shrinkage and selector operator (gLASSO) regularization method to control network sparsity [38]. Using the gLASSO regularization technique involves manually setting two parameters: the hyperparameter gamma (γ) and the tuning parameter lambda (λ). In following the tutorial provided by Epskamp and Fried [29], the hyperparameter was set conservatively (γ = 0.50). Setting γ conservatively reflects the extended BIC gLASSO regularization technique that prefers simpler models with fewer estimated edges. Additionally, in following Epskamp, Lunansky, Tio, and Borsboom [39], the tuning parameter was set modestly (λ = 0.01), reflecting a technique that limits spurious edges while retaining as many true edges as possible. 

Following the guidelines of Epskamp and Fried [29] and setting the parameters in this manner allowed resulting psychometric network models to be estimated with high specificity as is typical of gLASSO regularization; high sensitivity is also needed due to the reduction of false-positive edges typical when setting the tuning parameter low. The gLASSO regularization technique was followed to ensure the removal of estimated edges that were spurious (i.e., false-positive) or only occurring due to sampling error. This technique was only available for the psychometric network analyses conducted in qgraph as there currently is no way to specify these parameters using openMx.

## 3. Results

### 3.1. Data Preparation

Several variables were missing data in the H-WAIS-IV [7] dataset; when there was missing data values were imputed via multivariate imputation techniques provided by the mice [40] and VIM [41] packages in R^1^. The mice package imputes missing data using Markov Chain Monte Carlo methods on the correlation structure of the data. Using predictive mean matching as the imputation method, five datasets were generated for all missing data and a complete dataset was generated using the default method provided by the mice package.

### 3.2. Correlations, Descriptive Statistics, and Reliability

The correlation matrix for H-WAIS-IV data is presented in Table 1, with means and standard deviations presented in the bottom two rows. 

The H-WAIS-IV data demonstrated statistical reliability [42,43,44], with overall excellent internal consistency (Cronbach’s α = 0.94, 95% CI [0.93, 0.95]). Additionally, the internal consistency across the cognitive constructs present in the higher-order model of intelligence indicated excellent reliability for the construct representing crystallized intelligence (*G*_c_; α = 0.91, 95% CI [0.90, 0.92]); good reliability for the constructs representing visuospatial ability (*G*_v_; α = 0.82, 95% CI [0.80, 0.84]), working memory (*G*_wm_; α = 0.80, 95% CI [0.77, 0.82] ), processing speed (*G*_s_; α = 0.80, 95% CI [0.78, 0.82]), and acceptable reliability for the construct representing fluid reasoning (*G*_f_; α = 0.78, 95% CI [0.76, 0.81]).

### 3.3. Confirmatory Factor Analysis and Psychometric Network Models of the H-WAIS-IV

**Figure 2:**
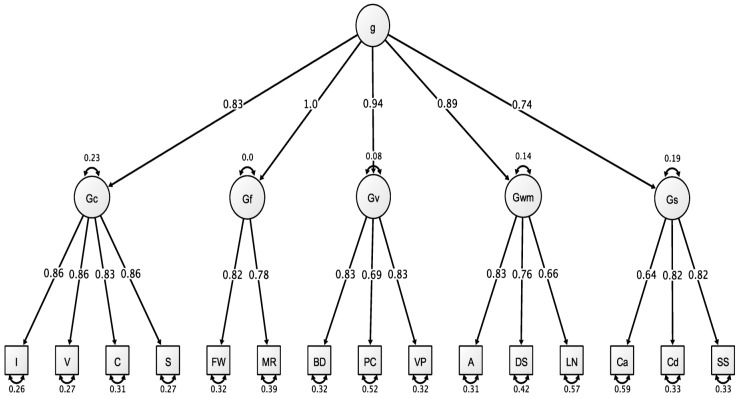
Hungarian Wechsler Adult Intelligence Scale Fourth Edition data applied to the Higher-Order model of intelligence. All values are standardized from the confirmatory factor analysis conducted using lavaan. Figure generated using Ωnyx.

Model fit indices for the confirmatory factor analyses and the psychometric network analyses are presented in Table 2. Direct model comparisons were not conducted because they would have been biased in favor of the psychometric network analyses. The network models conducted were exploratory in nature while the latent variable models were confirmatory. Thus, we caution readers from making direct comparisons based on the presented model fit indices. For a visualization of the H-WAIS-IV data fit to the higher-order latent variable structure, see Figure 2.

This figure can be interpreted as follows: (a) starting at the bottom, each H-WAIS-IV item has a uniqueness value in which larger values reflect greater unaccounted variance; (b) each first-order latent variable accounts for some degree of variance in items that is represented by the directional arrows connecting latent variables (*G*_c_, *G*_f_, *G*_v_ *G*_wm_, and *G*_s_) to items (represented by boxes); and (c) the general cognitive ability factor loadings onto each first-order latent variable is represented by the directional arrows connecting the superordinate *g* factor to first-order latent variables.

The latent variable models demonstrated varied fit across all reported fit indices. First, these latent variable models demonstrated unacceptable χ^2^ values and problematic values for the ratio between χ^2^ and degrees of freedom. However, the inflated χ^2^ is a general consequence of the large sample size and degrees of freedom associated with the higher-order model of intelligence and not completely representative of a problem with latent variable modeling techniques in general. Second, these latent variable models demonstrated values in the acceptable range for comparative fit indices (i.e., CFI and TLI) and RMSEA. It was also important to consider the measurement quality demonstrated by the standardized factor loadings presented in Figure 2. Across all H-WAIS-IV measures collected, the majority of standardized loadings surpassed the acceptable 0.70 level, with only three measures falling below this threshold. It is typical for model fit indices to demonstrate a negative correlation with measurement quality as measured by standardized factor loadings [45]. However, this trend did materialize in our latent variable models.

Unlike the latent variable models, the psychometric network models demonstrated excellent fit across most fit indices reported. First, both psychometric network models generated using qgraph and openMx demonstrated statistically significant χ^2^ values; however, the value of the ratio between χ^2^ and degrees of freedom for each was well below the value deemed acceptable. Second, the comparative fit indices (i.e., CFI and TLI) both demonstrated near perfect fit and RMSEA values were well below the 0.06 cutoff value used to measure model acceptability.

**Figure 3:**
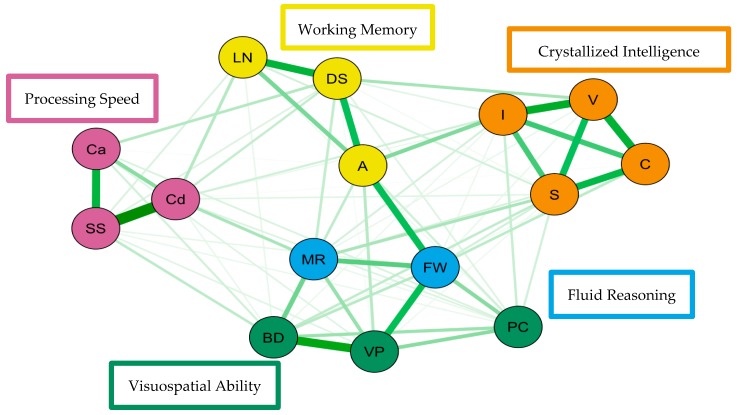
Weighted, undirected network model of the Hungarian Wechsler Adult Intelligence Scale Fourth Edition estimated using qgraph. Green edges indicate positive partial correlations.

**Figure 4:**
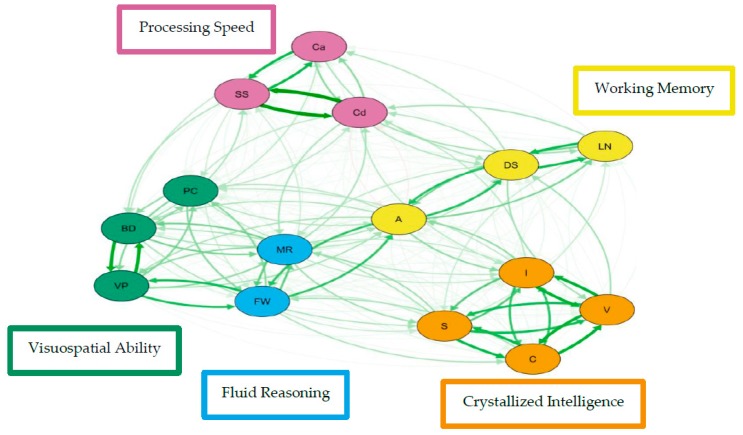
Weighted, directed psychometric network model of the Hungarian Wechsler Adult Intelligence Scale Fourth Edition estimated using openMx. Green edges indicate positive partial correlations and red edges indicate negative partial correlations between nodes.

For the visualization of these psychometric network models, see Figure 3 and Figure 4. 

For simplicity, nodes have been colored to reflect the latent structure of the higher-order latent variable measurement model and the width of each line represents the amount of association between pairs of nodes. The network models reveal four to five clusters of nodes. Three of the clusters are distinct, representing working memory, processing speed, and crystallized intelligence. The distinction between fluid reasoning and visuospatial ability is less clear. Also, in both network models, working memory and fluid reasoning are more central to the network than the other clusters. 

## 4. Discussion

The purpose of this study was to consider psychometric network analysis as an alternative approach to latent variable modeling to investigate the underlying structure of intelligence data measured by the H-WAIS-IV. Two competing theoretical perspectives were assessed whereby in one set of analyses a higher-order latent variable model was assumed; in a separate set of analyses, interconnected networks of overlapping processes or abilities were assumed. To this end, latent variable modeling and psychometric network analysis was applied to H-WAIS-IV data [7].

On the one hand, although theories of general intelligence like *g*-theory are compatible with reflective, higher-order latent variable models, they are incompatible with psychometric network analysis; these network models are estimated without *g* or any broad cognitive ability factors. The longstanding tradition in the psychological sciences of using structural equation modeling and other latent variable or factor analytic techniques is primarily due to the relative ease of using these statistical approaches. Additionally, these statistical procedures provide a useful technique for data reduction of complex data into fewer and, arguably, easier-to-comprehend factors or latent observations. On the other hand, theories of intelligence like POT that have previously been demonstrated to be compatible with formative, higher-order latent variable models [5] will always be incompatible with a reflective, higher-order latent variable model because, under POT, *g* is an emergent property or index. However, POT was compatible with psychometric network analysis and the network model description of H-WAIS-IV data. The psychometric network analysis and the network models presented here visually represent the positive manifold and the interaction between pairs of cognitive tests similarly to how POT proposes formative *g* and the explanation of the positive manifold via overlapping general-processes and specific-processes.

From a model fit perspective, we have corroborated the major findings presented by Kan et al. [8]: psychometric network models provided a better statistical description of H-WAIS-IV data than the traditional higher-order model established via confirmatory factor analysis. However, although consistent with Kan et al., a direct comparison between the psychometric network models and latent variable models would be inappropriate due to the different natures of these statistical techniques. Confirmatory factor analysis is a data-validation or confirmation technique and psychometric network analysis is a data-exploration technique. On the basis of the criteria provided by Schreiber et al. [31], when a reflective, higher-order latent variable model was applied to the H-WAIS-IV data, model fit indices were inconsistent with what would technically be deemed acceptable. This lack of model fit for the higher-order latent variable model of intelligence, a measurement model that is over 100 years old, warrants being accounted for. Descriptively, this trend provides evidence against *g* and *g*-theory in favor of an explanation of intelligence as an interconnected network of processes and abilities. This is in line with a formative view of *g* as the common consequence of correlations among observed variables in intelligence tests rather than the common cause. 

Psychometric network modeling techniques are new to the field of cognitive psychology and psychometrics. Network analysis can be used exploratively to determine variables that cluster together. Similar to exploratory factor analysis, this technique can be used as a precursor to using data-confirmation techniques. At times, latent variable models do not fit data as well as theorized or hypothesized. In cases like this, network modeling can be used to visualize and assess the one-to-one relationships among observed variables that might illuminate reasons for poor model fit [46]. Additionally, future research that employs network modeling must consider network stability and the development of confirmatory network modeling techniques. First, guidelines provided by Epskamp, Borsboom, and Fried [47] regarding psychometric network stability analyses are available for researchers with complete, raw datasets. This procedure employs bootstrapping techniques to assess the overall accuracy and invariance of network models while allowing researchers to establish confidence intervals on an edge-by-edge basis. These confidence intervals can be used to determine the accuracy of each estimated edge in the network. Edges are estimated accurately when the confidence intervals surrounding them are relatively small. However, these confidence intervals do not function as an assessment of whether the edge weight significantly differs from zero. Additionally, due to the nature of confidence intervals, edges can be directly compared using a nonparametric difference test to determine whether an edge in the network demonstrates statistically significant differences from other estimated edges. Thus, stability analyses could be used to describe whether network models are generalizable and invariant across datasets. Second, many researchers interested in psychometric network modeling have begun to recognize the necessity of confirmatory network modeling approaches that could be used as a data-validation technique to the exploratory nature of psychometric network analysis.

Finally, researchers employing latent variable modeling techniques ought to consider the questions proposed by Borsboom et al. [15] (pp. 204) concerning latent variables:

Should we assume that the latent variable signifies a real entity or conceive of it as a useful fiction, constructed by the human mind? Should we say that we measure a latent variable in the sense that it underlies and determines our observations, or is it more appropriately considered to be constructed out of the observed scores? What exactly constitutes the relation between latent variables and observed scores? Is this relation of a causal nature? If so, in what sense? And, most important, is latent variable theory neutral with respect to these issues?

Additional analyses of large-scale data sets applying both psychometric network modeling and latent variable models might further reveal the advantages of each approach. As a recent development, latent variable network modeling [48] combines the two approaches by describing a network of connections between latent variables that account for performance of tests of cognitive abilities. This might reconcile the two approaches, bringing the “best of both worlds” to research as to the structure of human cognitive abilities.

## Figures and Tables

**Table 1:** Correlation matrix and descriptive statistics of Hungarian Wechsler Adult Intelligence Scale Fourth Edition (H-WAIS-IV).

	I	V	C	S	PC	BD	FW	MR	VP	A	DS	LN	Ca	Cd	SS
I	1.00	0.75	0.71	0.72	0.51	0.55	0.59	0.58	0.53	0.60	0.54	0.46	0.34	0.49	0.39
V		1.00	0.73	0.73	0.44	0.49	0.56	0.55	0.49	0.52	0.51	0.41	0.31	0.46	0.39
C			1.00	0.72	0.44	0.51	0.57	0.55	0.48	0.52	0.47	0.40	0.30	0.44	0.36
S				1.00	0.50	0.57	0.60	0.55	0.54	0.55	0.52	0.45	0.34	0.48	0.38
PC					1.00	0.55	0.57	0.50	0.56	0.52	0.45	0.38	0.36	0.43	0.41
BD						1.00	0.63	0.62	0.70	0.58	0.48	0.43	0.41	0.49	0.48
FW							1.00	0.64	0.68	0.66	0.51	0.44	0.35	0.47	0.45
MR								1.00	0.61	0.57	0.51	0.41	0.35	0.50	0.44
VP									1.00	0.59	0.49	0.41	0.39	0.44	0.45
A										1.00	0.62	0.52	0.37	0.42	0.42
DS											1.00	0.57	0.34	0.49	0.45
LN												1.00	0.31	0.44	0.40
Ca													1.00	0.49	0.54
Cd														1.00	0.67
SS															1.00
*M*	9.99	9.99	10.00	9.98	10.03	9.99	9.99	10.00	9.99	9.99	10.00	9.30	10.05	9.99	9.98
*SD*	2.94	2.98	3.00	2.99	2.97	3.00	2.97	2.99	2.98	3.02	2.98	3.45	2.98	2.94	2.99

Note. I = information; V = vocabulary; C = comparisons; S = similarities; PC = picture completion; BD = block design; FW = figure weights; MR = matrix reasoning; VP = visual puzzles; A = arithmetic; DS = digit span; LN = letter-number sequencing; Ca = cancellation; Cd = coding; SS = symbol search. N = 1,112.

**Table 2:** Model Fit Indices for Latent Variable and Network Models of Hungarian Wechsler Adult Intelligence Scale-Fourth Edition Data.

	Models	χ^2^	*df*	CFI (TLI)	RMSEA	AIC	BIC
lavaan/qgraph	WAIS-IV CFA	376.44 ***	85	0.97 (0.97)	0.06	528.99	529.91
	WAIS-IV Network	48.56 *	33	1.00 (1.00)	0.02	211.52	212.44
openMx	WAIS-IV CFA	389.21 ***	85	0.97(0.97)	0.06	459.21	523.53
	WAIS-IV Network	50.50 *	33	1.00(0.99)	0.02	224.50	384.37

Note. *** *p* < 0.001; * *p* < 0.05; χ^2^ = Model chi-square value; *df* = degrees of freedom; AIC = Akaike information criteria; BIC = Sample size adjusted Bayesian information criteria; RMSEA = Root mean square error of approximation; CFI = Comparative fit index; TLI = Tucker-Lewis index. To make AIC and BIC values comparable, qgraph values were transformed by dividing each value by a product of two and the number of estimated parameters.

## Footnotes

1 Missing data was present for more than 15% of the sample for (a) Letter-Number Sequencing, (b) Figure Weights, and (c) Cancellation. Prior to implementing the imputation techniques, the sample size was 875. Following imputation techniques the sample size increased to 1112 subjects.
